# Value of FDG-PET/MR in Oral Focus Assessment in Head and Neck Cancer Patients—A Feasibility Study

**DOI:** 10.3389/fmed.2022.809323

**Published:** 2022-03-21

**Authors:** Silvio Valdec, Fabienne A. Bosshard, Martin Hüllner, Dominic R. Schwaninger, Larissa Stocker, Barbara Giacomelli-Hiestand, Bernd Stadlinger

**Affiliations:** ^1^Clinic of Cranio-Maxillofacial and Oral Surgery, Center of Dental Medicine, University of Zurich, Zurich, Switzerland; ^2^Division of Periodontology, Department of Stomatology, Dental School, University of São Paulo, São Paulo, Brazil; ^3^Department of Nuclear Medicine, University Hospital Zürich, University of Zurich, Zurich, Switzerland; ^4^Clinic of Orthodontics and Pediatric Dentistry, Center of Dental Medicine, University of Zurich, Zurich, Switzerland

**Keywords:** head and neck cancer, radiation therapy, dental focus, positron emission tomography–magnetic resonance imaging, periapical radiography, panoramic radiography

## Abstract

FDG-PET/MR is a hybrid imaging modality used for the staging and restaging of advanced head & neck cancer (HNC) patients. Their treatment typically involves radiation therapy, which requires previous dental focus assessment. The aim of this study was to analyze if staging FDG-PET/MR is a valuable tool for oral focus assessment. For this purpose, FDG-PET/MR findings, such as metabolic activity of periapical radiolucencies and marginal periodontitis, were retrospectively compared with conventional standardized dental focus assessment, including dental radiographs and clinical assessment of 124 teeth in seven patients. Increased FDG uptake of periapical lesions was found in one out of 23 lesions. Increased FDG uptake of the marginal periodontium was recorded in one out of 34 lesions. In summary, standardized dental focus assessment by panoramic radiography and periapical radiographs may be enriched by information from FDG-PET/MR, showing active inflammation in dental foci. However, many dental foci have no correlate in FDG-PET/MR. The treatment decision for oral foci may benefit from the visualized presence or absence of metabolic activity on FDG-PET/MR.

## Introduction

Positron emission tomography/magnetic resonance (PET/MR) imaging using the radiotracer 18F-fluorodeoxyglucose (FDG-PET/MR) is a hybrid imaging modality, which is mainly used in oncological patients for staging and restaging purposes (1). However, it may also be used for imaging inflammation and infection (2).

Head & neck cancer (HNC) is the seventh most common cancer worldwide, with half a million new diagnoses per year (3, 4). In Switzerland, more than 1,000 new HNC cases are diagnosed each year, reverting to a lifetime HNC risk of 0.7% in women and 1.6% in men (5).

In advanced HNC, treatment typically involves radiation therapy with or without surgery and chemotherapy (4, 6, 7). This treatment harbors several short-term and long-term complications owing to tissue damage from ionizing radiation. Oral infection or inflammation is a known risk factor for such radiation-induced oral damages (8). Hence, it is highly recommended that patients undergo oral health screening, including clinical and radiological examination, to detect potential foci requiring treatment before the commencement of radiation therapy (4, 9, 10).

Panoramic radiography (OPT) serves as a standard radiological assessment for hard tissue pathologies. Its advantages are comparably low radiation exposure, widespread availability, and good image quality. OPT is mostly supplemented by periapical radiographs in selected cases, such as root canal treated teeth. After incidental findings, three-dimensional imaging such as cone beam computed tomography (CBCT) or MR can also be performed during the initial examination (11). Further, a thorough oral examination is performed. After dental focus assessment, any acute or potential inflammatory condition diagnosed, such as marginal and apical periodontitis, will be treated (12). The patient remains in dental care during and after radiotherapy or chemotherapy (10, 13). While dental focus assessment is not a reimbursed indication for FDG-PET imaging in Switzerland, dental foci are sometimes discovered incidentally on staging / restaging examinations of head and neck cancer patients.

At our institution, every HNC patient requiring radiation therapy undergoes either whole-body positron emission tomography/computed tomography (PET/CT) or PET/MR using the radiotracer 18F-fluorodeoxyglucose (FDG).

The aim of our study was to find out whether FDG-PET/MR offers added value in dental focus assessment. To the best of our knowledge, this is the first study analyzing the added value of FDG-PET/MR in dental focus assessment.

## Materials and Methods

### Patient Selection

HNC patients who underwent FDG-PET/MR for staging and standardized dental focus assessment prior to radiation therapy at the University Hospital of Zurich between December 2016 and December 2018 were included into this study. FDG-PET/MR was conducted at the Department of Nuclear Medicine at the University Hospital Zürich, Switzerland. Dental focus assessment was performed at the Clinic of Cranio-Maxillofacial and Oral Surgery at the Center of Dental Medicine, University of Zurich. This study was approved by the local ethics committee of Zürich (Nr. 2017-01378).

Only patients with signed consent for the use of their medical data for research were included. Other inclusion criteria were scheduled radiotherapy with or without surgery and/or chemotherapy, and the availability of a FDG-PET/MR exam including a diagnostic head and neck MR protocol, as well as availability of panoramic radiography (OPT) and periapical X-rays. Only patients with a maximum time interval of 3 months between these exams, without any surgical or therapeutic intervention in between, were included. Patients with blurred radiographic images were excluded. Image angulations were ignored.

### Image Acquisition

PET/MR image acquisition was carried out as described previously in detail (14).

A BMI-adapted body weight-dependent FDG dosage protocol was used (15). A Dixon-type MR pulse sequence was used for attenuation correction (16, 17). In brief, the MR protocol consisted of the following MR pulse sequences: Axial 2-point Dixon-type sequence and coronal T2-weighted sequence with fat suppression for the whole-body; axial respiration-triggered T2-weighted sequence for the lung and upper abdomen; regionalized head and neck axial and coronal T2-weighted sequence with fat suppression, axial T1-weighted sequence without gadolinium-based contrast and without fat suppression, axial, coronal and sagittal T1-weighted sequences with gadolinium-based contrast and with fat suppression.

Every dental focus assessment included a recording of radiographic findings. Panoramic radiography (OPT), periapical radiographs of every root canal-treated tooth and bite wings for caries evaluation were taken and archived in the PACS (Synedra, Apollon Innsbruck, Austria). OPTs were generated in a standardized position, using Cranex 3D (Soredex, KaVo, Biberach, Germany). Periapical radiographs were generated using Heliodent DS (Dentsply-Sirona, Bensheim, Germany). The intraoral X-ray was operated at 60 kV and 7 mA. Parallel technique was used, with a focus-patient distance of approximately 21 cm.

### Image Analysis

The analysis of the X-rays, generated during the standardized dental focus assessment, was conducted by board-certified dentists (LS, DS) under the supervision of an oral surgeon (BGH). In case of disagreement, a consensus decision was reached by discussing the case in detail (LS DS, BGH and BS). All dental X-rays were analyzed in DICOM format using Synedra Viewer (Synedra, Apollon Innsbruck, Austria) under standardized conditions on a diagnostic monitor (NEC, MDview 243). FDG-PET/MR images were analyzed using a dedicated review workstation (AW 4.6, GE Healthcare) (MH). All imaging modalities (FDG-PET/MR, OPT, dental X-rays) were analyzed separately.

### Analyzed Radiological Parameters

For radiological evaluation, the focus was set on two main parameters: periapical lesions and marginal bone level. These predefined parameters were assessed on the X-rays acquired during the standardized dental focus assessment (OPT, periapical radiographs and bite wings) and on FDG–PET/MR (except marginal bone level). On FDG-PET/MR, increased metabolic activity related to dental lesions was recorded as presence (expressed as SUV_max_) or absence. Finally, all teeth were examined for root canal fillings.

### Periapical Lesions

For the classification of the periapical lesions, the periapical index (PAI) described by Orstavik et al. (18) was used. This index ranges from 1 (healthy) to 5 (severe, exacerbating apical periodontitis). In addition, the size of periapical lesions was recorded (smaller or larger than 5 mm in diameter).

### Marginal Periodontium

For the classification of the marginal bone level, the marginal periodontitis index (MPI) described by Kito et al. was applied (19). This index distinguishes 4 sections (1–4) and estimates the physiological bone level compared to the actual bone level. Bone loss of less than one-third was classified as “1”, one-third up to half as “2”, half up to two-thirds as “3”, and more than two thirds as “4”. A marginal bone lesion was defined as MPI score ≥2.

### Clinical Outcome Parameter

#### Percussion Sensitivity

In addition to radiological data, the clinical parameter percussion sensitivity was extracted from the standardized patients' charts used at our institution. All teeth with periapical lesions were analyzed for their percussion sensitivity. Findings were noted as either 1 (sensitivity present) or 0 (sensitivity absent).

### Statistics

Total number of patients, demographic data and the outcome measurements were recorded using Microsoft Excel. A descriptive analysis was performed for all analyzed parameters (PAI Score, PL, percussion sensitivity, MPI Score, MP, SUV_max_). Tables were produced for data representation.

## Results

During the study period of 24 months, a total of 13 patients with diagnosed HNC underwent FDG-PET/MR for staging and dental focus assessment. Six of these patients were excluded because the required time interval of 3 months or less between FDG-PET/MR and dental focus assessment was not met. Thus, the final study population consisted of a total of seven patients (one woman and six men). The median age was 72 (23–82 years). The median time difference between the FDG-PET/MR scan and the dental focus assessment was 3 weeks (2–11 weeks). A total of 124 teeth were analyzed for dento-alveolar parameters.

### Periapical Lesions

**Figure 1:**
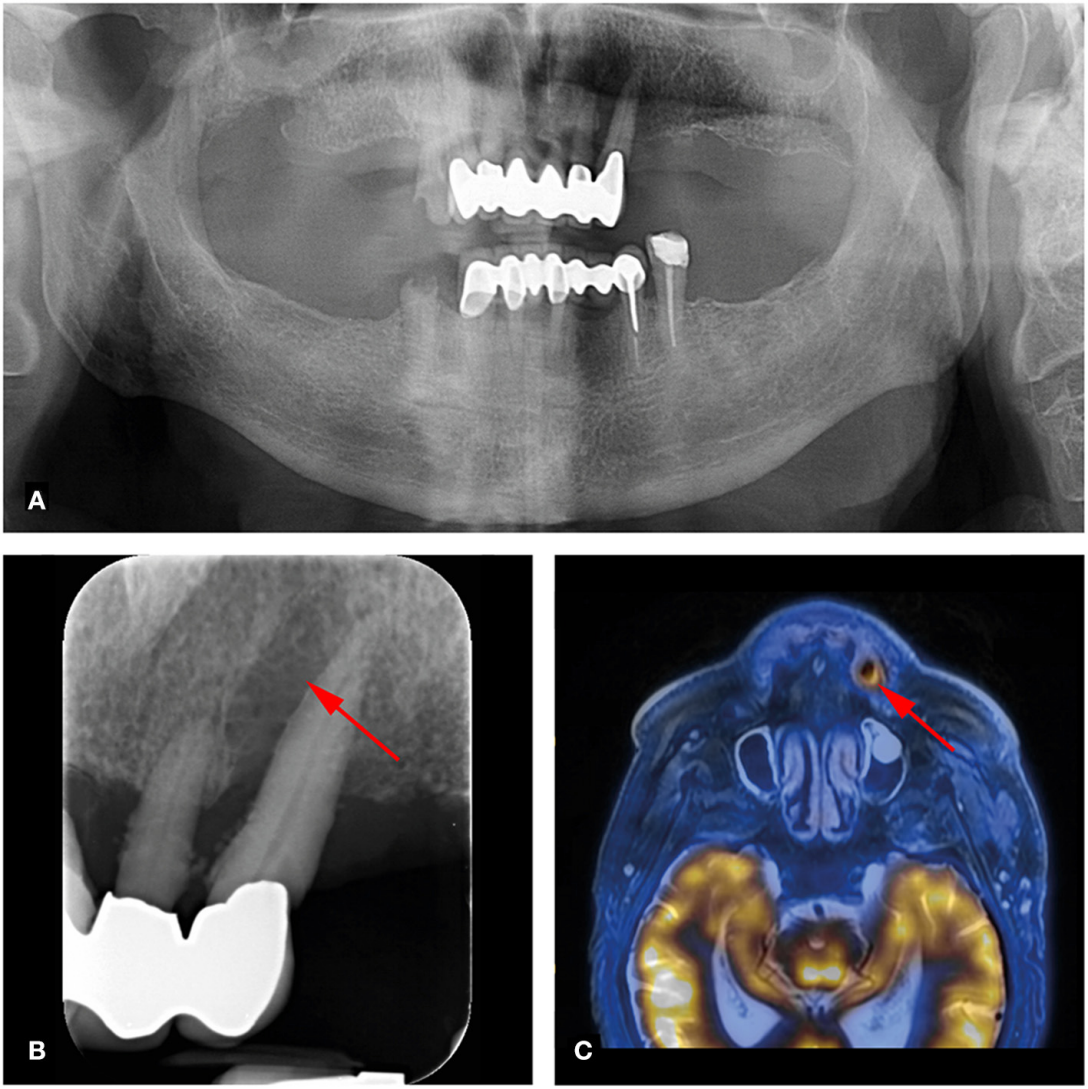
73-year-old man with right-sided hypopharynx carcinoma cT3 cN2b cM0. Panoramic **(A)** and dental radiography **(B)** shows a periapical lesion with marginal bone loss in region 23 (arrow; PAI score 4, MPI score: 4). **(C)** FDG-PET/MR shows a metabolically active osteolysis at tooth 23 with a SUVmax of 6.8 (arrow).

**Figure 2:**
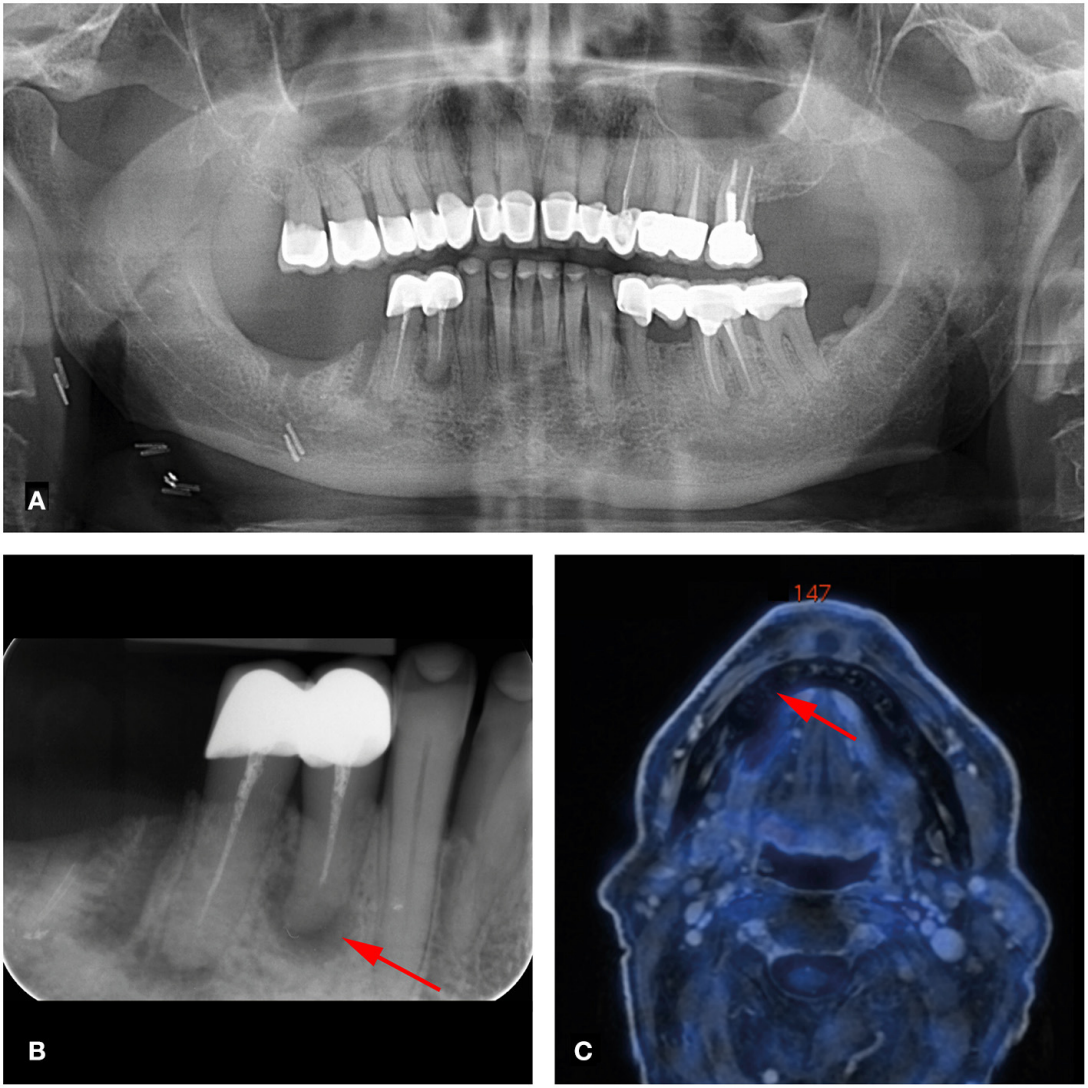
66-year-old man with right-sided oral cavity squamous cell carcinoma cT1 cN2b cMx. Panoramic **(A)** and dental radiography **(B)** shows a periapical lesion of tooth 44 of less than 5mm (arrow; PAI score 4, MPI score 1). **(C)** FDG-PET/MR shows no increased metabolic activity of this lesion (arrow).

Of the 124 analyzed teeth, 23 (18.5%) showed periapical lesions (PAI ≥ 2) on dental radiographs (OPT/periapical X-rays). The PAI score ranged from 1 to 4, with a mean of 1.49 ± 1.06. Two periapical lesions were larger than 5 mm. On FDG-PET/MR, 19 of the 23 (82.6%) periapical lesions were detected. PET/MR did not detect any additional periapical lesions. Clinical data revealed a total of three percussion sensitive teeth. Increased FDG uptake of periapical lesions was recorded in one out of 19 lesions with an SUV_max_ of 6.8. This lesion showed a large marginal and apical bone resorption with a PAI score of 4 (Figure 1). This tooth showed no percussion sensitivity (Table 1). The other 18 teeth with apical lesions did not show increased FDG uptake, as illustrated in Figure 2 and Table 1.

**Table 1:** Periapical lesions (PL) in dental X-rays and FDG-PET/MR.

**PAI Score**	**PL in dental radiographs (*n =* 23)**	**PL in PET/MR (*n =* 19)**	**PL with increased SUV_**max**_ (*n =* 1)**	**PL in dental radiographs >5mm (*n =* 2)**	**PL in dental radiographs <5mm (*n =* 18)**	**Percussion sensitivity**
1	0	0	0	0	0	2
2	1	0	0	0	0	0
3	6	3	0	0	4	1
4	16	16	1	2	14	0

### Marginal Periodontium

90 out of 124 Teeth (72.6%) showed an MPI score of 1 (marginal bone loss of less than one third compared to the physiological bone level) in dental radiographs. All marginal lesions (definition: MPI score ≥ 2) were visualized on dental radiographs. For these 34 marginal lesions, increased FDG uptake on FDG-PET/MR was seen in only one tooth (SUV_max_ 6.8) (Table 2). Images of this patient with increased FDG uptake of a periapical lesion and its marginal periodontium are shown in Figure 1.

**Table 2:** Marginal periodontium (MP) in dental X-rays and FDG-PET/MR.

**MPI score**	**MP (*n =* 124)**	**MP in dental radiographs (*n =* 54)**	**MP with increased SUV_**max**_ (*n =* 1)**
1	90	20	0
2	16	16	0
3	12	12	0
4	6	6	1

## Discussion

The aim of our study was to investigate the added value of FDG-PET/MR in oral focus assessment of HNC patients. Dental radiographs, showing periapical and/or marginal periodontal lesions were compared to FDG-PET/MR. We analyzed whether metabolic activity on FDG-PET/MR correlates with findings on radiographs and clinical percussion data.

A total of 124 teeth in seven patients were examined for dento-alveolar parameters. Only one apical/marginal periodontal lesion showed increased FDG uptake with an SUV_max_ of 6.8. In contrast, another patient with a huge periapical lesion at tooth 44 (>5mm, PAI = 4) who further had no marginal bone loss (MPI = 1) showed no FDG uptake on FDG-PET/MR (Figure 2). While 23 apical lesions were detected on dental radiographs, FDG-PET/MR detected only 19 of these (82.6%).

Numerous studies have investigated the detection of apical lesions. Imaging modalities used include dental radiographs, ultrasound, and dental MR (20–22). A recent systematic review showed that ultrasound can distinguish periapical lesions better compared with dental radiographs, although dental radiographs still represent the gold standard (20). There is also a deep learning algorithm that surpasses experienced oral surgeons in the detection of periapical lesions in dental radiographs (22). However, the interpretation of the degree of inflammation of periapical lesions remains unclear. In dental radiographs, it could not be distinguished between apical granulomas and radicular cysts after evaluation of correlating histopathologic examinations (23). However, a recent study proofed that this differentiation is possible with MR (21). Nevertheless, interpretation of apical lesion clinical activity remains a challenge (24). In our study, most of the apical lesions as well as the marginal periodontal lesions did not show signal uptake.

Metabolic activity of potential oral foci cannot be determined on dental radiographs. Presence of metabolic activity, however, will contribute to a treatment decision. Active, presumably acute foci should be treated immediately, while inactive, presumably chronic foci may be treated in the later course under specific circumstances (12, 25). Another point in decision making, certainly is dynamic over time. For instance, radiotherapy or immunosuppression may transform a chronic, inactive lesion into an acute lesion.

In our study, no association between signs of inflammation on dental radiographs, clinical percussion data and increased FDG uptake on FDG PET/MR was found. Today, few studies have investigated the correlation of FDG PET/CT and oral foci. A retrospective study by Dijkstra et al. investigated endocarditis patients who underwent FDG-PET/CT. In their study, also no correlation between oral cavity PET findings and inflammation/infection was found (26). Nevertheless, the authors recommend further investigation to determine whether FDG-PET/CT imaging may proof useful for diagnosing inflammation and infection in the oral cavity (26). In our study, positive percussion sensitivity was not associated with increased metabolic activity on FDG-PET/MR.

Another study by Kito et al. demonstrated a correlation between FDG uptake and inflammatory extent of apical and periodontal lesions in 44 patients (19). Yamahiro et al. detected FDG uptake in different acute periodontal foci, whereas in chronic infection no increased FDG uptake was found. The authors concluded that FDG-PET/CT may serve as a valid tool to detect acute oral infections in high-risk patients (27).

In HNC imaging, FDG-PET/MR is a promising modality as it simultaneously provides morphological, functional, and molecular information (2, 7, 28, 29). In this respect, it may be expected that further studies will investigate the added value of FDG-PET/MR also in oral focus examinations in the future.

The main limitation of our study is its comparably small sample size, limiting the generalization of the results. Another limitation is the time interval between FDG-PET/MR and standardized focus assessment, which was up to 3 months, possibly resulting in changes of lesions during this time. Further prospective studies including follow-up data are desired to gain more information on the added value of FDG-PET/MR in dental diagnostics.

## Conclusion

While FDG-PET/MR detected a certain percentage of periapical lesions, no association was found between FDG uptake and the degree of inflammation of apical lesions and marginal bone loss. Future studies with larger cohorts should determine if FDG-PET/MR results shall be considered by dentists carrying out oral focus assessment of HNC patients.

## Data Availability Statement

The raw data supporting the conclusions of this article will be made available by the authors, without undue reservation.

## Ethics Statement

The studies involving human participants were reviewed and approved by Ethics committee of Zürich (No. 2017-01378). The patients/participants provided their written informed consent to participate in this study.

## Author Contributions

BS and MH conceived the idea. MH took responsibility of the FDG-PET MR part. LS, DS, and BG-H were involved in the planning and did the data collection. BG-H and BS supervised the data collection. Data analysis was performed by FB and SV. BS supervised the findings of this work. FB and SV created the manuscript. BS and MH edited the manuscript. All authors approved the final manuscript.

## Conflict of Interest

MH received research grants from GE Healthcare, grants from the CRPP Artificial Intelligence in oncological Imaging Network by the University of Zurich, and a fund by the Alfred and Annemarie von Sick legacy for translational and clinical cardiac and oncological research. The remaining authors declare that the research was conducted in the absence of any commercial or financial relationships that could be construed as a potential conflict of interest.

## Publisher's Note

All claims expressed in this article are solely those of the authors and do not necessarily represent those of their affiliated organizations, or those of the publisher, the editors and the reviewers. Any product that may be evaluated in this article, or claim that may be made by its manufacturer, is not guaranteed or endorsed by the publisher.
